# Effect of infrared irradiation combined with mannitol and kinesiology tape on postoperative swelling and pain in patients with a periarticular ankle fracture

**DOI:** 10.12669/pjms.39.1.5869

**Published:** 2023

**Authors:** Yuanyuan Liu, Deli Guo, Xinghua Zhou, Bo Wang, Panxiang Li, Tao Li

**Affiliations:** 1 Yuanyuan Liu, Department of Orthopedics, Baoding NO.1 Central Hospital, Baoding, Hebei, 071000, China; 2 Deli Guo, Department of Orthopedics, Baoding NO.1 Central Hospital, Baoding, Hebei, 071000, China; 3 Xinghua Zhou, Department of Orthopedics, Baoding NO.1 Central Hospital, Baoding, Hebei, 071000, China; 4 Bo Wang, Department of Orthopedics, Baoding NO.1 Central Hospital, Baoding, Hebei, 071000, China; 5 Panxiang Li, Department of Orthopedics, Baoding NO.1 Central Hospital, Baoding, Hebei, 071000, China; 6 Tao Li, Department of Orthopedics, Baoding NO.1 Central Hospital, Baoding, Hebei, 071000, China

**Keywords:** Periarticular ankle fracture, Infrared ray, Mannitol, Kinesiology tape, Swelling

## Abstract

**Objectives:**

To explore the effect of infrared irradiation combined with mannitol and Kinesiology tape on postoperative swelling and pain in patients with a periarticular ankle fracture.

**Methods:**

The research subjects of this study were 88 patients with periarticular ankle fracture treated by surgery in the Department of Orthopedics of Baoding No.1 Central Hospital from October, 2019 to May, 2021. They were randomly divided into the observation group and the control group based on the random number table method, with 44 cases in each group. All patients were treated after the operation. Patients in the control group were treated with conventional drugs; while those in the observation group were provided with infrared irradiation combined with mannitol and Kinesiology tape. Further comparison was conducted on the degree of swelling, pain and satisfaction after treatment at three, five and seven days after operation.

**Results:**

At three, five and seven days after operation, the cross-section diameter of the injured limb was significantly smaller in the observation group than that in the control group, and the difference was statistically significant (*p<*0. 05). The degree of pain in both groups was significantly lower at three, five and seven days after operation than that before treatment; moreover, the degree of pain in the observation group was significantly lower than that in the control group, and the difference was statistically significant (*p<*0. 05). Besides, the comparison of posttreatment satisfaction in both groups after treatment revealed that the total satisfaction of patients in the observation group (97.73%) was higher than that in the control group (79.55%), with a statistically significant difference (*p<*0.05).

**Conclusion:**

Infrared irradiation combined with mannitol and Kinesiology tape can effectively alleviate postoperative swelling and pain of patients with a periarticular ankle fracture.

## INTRODUCTION

The ankle joint of a human is composed of the lower end of tibiofibular and talus. There is a high risk of fracture when it is injured by violence. According to the sources of external force and locations of injury to the ankle, there may be different types and degrees of fractures, which are referred to as periarticular ankle fractures collectively.^1^ Periarticular ankle fractures are common in daily life and account for about 7.6% of fractures among adults, and the fractured patients may have ankle pain, swelling, varus or valgus deformity, difficulties in movement, etc.^2^ Swelling after fracture surgery may be explained by various causes. In addition to the damage of limb fracture itself to the soft tissue to some extent, there may be secondary damage to the soft tissue intraoperatively, and swelling can also be induced owing to the blood circulation dysfunction at the site of ankle fracture; moreover, swelling, pain and other symptoms will be aggravated in case of wound infection during postoperative recovery.^3^ Swelling may hinder the postoperative recovery of surgical patients. In the absence of timely treatment for serious swelling, complications are likely to show, such as osteofascial compartment syndrome or traumatic arthritis. Therefore, it is necessary to make active treatment once patients have swelling and pain.^4^ At present, drug therapy of mannitol combined with physical therapy has been accepted as the conventional treatment for postoperative swelling and pain in patients with a periarticular ankle fracture. However, this therapy has the disadvantages of delayed time to achieve the subsidence of swelling and hence poor curative effect. While infrared ray can promote blood circulation and is beneficial for the alleviation of soft tissue edema. In addition, Kinesiology tape is a type of elastic adhesive tape, which can relieve pain and promote circulation. In order to reduce postoperative swelling and pain, this study intended to explore the effect of infrared irradiation combined with mannitol and Kinesiology tape.

## METHODS

The research subjects were 88 patients with periarticular ankle fracture treated by surgery in the Department of Orthopedics of Baoding NO.1 Central Hospital from October, 2019 to May, 2021. According to the random number table method, they were randomly divided into the observation group and the control group, with 44 cases in each group. As shown in Table-I, there was no significant difference in the comparison of general data between the two groups (*p>*0.05). The study was approved by the Institutional Ethics Committee of Baoding NO.1 Central Hospital (No.:2020033; May 21, 2021), and written informed consent was obtained from all participants.

**Table-I:** Comparison of general data between the two groups.

Groups	Cases	Gender (M/F)	Age (years)
Observation group	44	24/20	55.45±20.05
Control group	44	23/21	54.94±19.85
χ²/t	-	0.046	0.120
*P* value	-	0.831	0.905

### Inclusion criteria:

- Patients who met the diagnostic criteria of periarticular ankle fracture and were diagnosed by X-ray and CT;
- patients with normal cognitive function;
- patients hospitalized within 12 hours after injury.

### Exclusion criteria:

- Patients with open fractures;
- Patients with fractures in other parts;
- Patients allergic to infrared ray, mannitol, or materials of Kinesiology tape;
- Patients who did not cooperate with medical advice and had poor compliance.

All patients were treated after ankle surgery. Patients in the control group were treated with conventional drugs, while those in the observation group were provided with infrared irradiation combined with mannitol and Kinesiology tape.

### Control Group:

The patients in this group were treated with conventional drugs using 20% Mannitol Injection (national medicine permission number [NMPN] H20033039, Zhejiang Tianrui Pharmaceutical Co., Ltd.) for five days (twice a day, and 125 ml per time).

### Observation Group:

The patients in this group were provided with infrared irradiation combined with mannitol and Kinesiology tape for the same period as the control group. *Infrared irradiation:* The treatment was performed using QX-735 medical vertical cart far-infrared therapeutic apparatus (Guangzhou QiaoXin Medical Device Technology Development Co., Ltd.). During treatment, patients were informed to keep their supine position, with the affected limb padded up, and the spotlight cap adjusted according to the patient’s physical condition until it was comfortable. The power range was 700-1000 W; the irradiation distance was 30-50 cm (twice a day for 0.5 h each time). *Drug therapy:* Drug therapy was conducted with the use of 20% Mannitol Injection (NMPN H20033039, Zhejiang Tianrui Pharmaceutical Co., Ltd.) for five days (twice a day intravenously, and 125 ml per time). *Kinesiology tape:* With the use of Guardee Kinesiology tape (Suzhou Medsport Products Co., Ltd.), the tape was cut into a shape (I-shape, Y-shape, X-shape, etc.) with appropriate length and fit the patient’s ankle. Before applying the tape, the skin was depilated and cleaned, disinfected with an alcohol prep pad to avoid the wound incision, and the tape was applied on the ankle after drying to keep the ankle in the original physiological state. Both ends of the tape were torn off without stretching the area, with one side torn off and applied to the upper bundle of the left muscle of the left ankle joint (anchor point). After unfolding with natural tension, the tape was applied to the moving point. After application, the tape was rubbed and swept repeatedly several times to make the tape more stable and fit the skin. The tape should be changed in the hospital within 48-72 hours to prevent the weakening of elasticity and adhesion of skin secretions. Special attention should be paid to cleaning the skin before replacement. Five times constituted a course of treatment.

### Observational Indexes:

Further comparison was conducted in all patients focusing on the degree of swelling, pain and satisfaction after treatment at three, five and seven days after the operation. *Evaluation of swelling degree:* The cross-section diameter of the connecting line between the medial and lateral malleolus of the injured limb was measured by using the same tape measure, once in the morning, noon and evening every day, with the measurement of the average value for evaluation *Evaluation of pain degree:* Visual analog scale (VAS) was used to evaluate the degree of pain. A straight line of 10 cm in length was drawn on a piece of white paper. Each 1cm interval represented different pain degrees (1-10 points from low to high). Patients were informed to mark on the straight line according to their current feeling of pain degree. A higher score might indicate a higher degree of pain. *Evaluation of treatment satisfaction*: All patients were investigated and recorded through the self-made satisfaction questionnaire in our hospital. The grades were very satisfactory, satisfactory and dissatisfactory. The degree of satisfaction was expressed as the rate (%), and total satisfaction was calculated according to the formula of (very satisfactory + satisfactory) /n*100% (n, case number).

### Statistical Analysis:

Statistical analysis was realized by using SPSS22.0 software. The measurement data were expressed in (*χ̄*±*S*), and the independent sample t-test or analysis of variance of repeated measurement design was used for data comparison between groups. The counting data were presented in percentage (n, %), and compared using chi-square (c²) test with cross table. P<0.05 was considered statistically significant.

## RESULTS

As shown in Table-II, at three, five and seven days after operation, the cross-section diameter of the injured limb was significantly smaller in the observation group than that in the control group, and the difference was statistically significant (*p<*0. 05).

**Table-II:** Comparison of the cross-section diameter of the injured limb between the two groups (cm, *χ̄*±*S*)

Groups	Cases	3d after operation	5d after operation	7d after operation
Observation group	44	25.03±2.19	20.51±2.03	18.51±0.64
Control group	44	24.95±2.21	28.69±2.15	25.69±2.61
*F value*	-	F_time points_=135.907, F_groups_=177.631, F_interactive_=280.799		
*P value*	-	P_time points_<0.001, P_groups_<0.001, P_interactive_<0.001		

Based on Table-III, the degree of pain in both groups was significantly lower at three, five and seven days after operation than that before treatment; moreover, the degree of pain in the observation group was significantly lower than that in the control group, and the difference was statistically significant (*p<*0. 05).

**Table-III:** Comparison of postoperative pain between the two groups (points, *χ̄*±*S*)

Groups	Cases	T1	T2	T3
Observation group	44	7.03±1.19	6.51±1.24	3.22±0.60
Control group	44	7.11±1.21	6.69±1.57	5.79±1.24
*F value*	-	F_time points_=254.565, F_groups_=18.950, F_interactive_=67.811		
*P value*	-	P_time points_<0.001, P_groups_<0.001, P_interactive_<0.001		

According to the posttreatment satisfaction in both groups after treatment (Table-IV), the total satisfaction of patients in the observation group (97.73%) was higher than that in the control group (79.55%), with a statistically significant difference (*p<*0.05).

**Table-IV:** Comparison of satisfaction between the two groups (n, %).

Groups	Cases	Very satisfied	Satisfied	Dissatisfied	Satisfaction (%)
Observation group	44	32 (72.73)	11 (25)	1 (2.27)	43 (97.73)
Control group	44	10 (22.73)	25 (56.82)	9 (20.45)	35 (79.55)
χ² value	-	-	-	-	7.221
*P* value	-	-	-	-	0.007

## DISCUSSION

Ankle joint, also known as the talocrural joint, comprises a tibia, distal fibula and talus that make up the bone of the lower leg, which is the largest load-bearing joint in human body.^5^ Periarticular ankle fractures account for about 3.92% of systemic fractures, ranking first in intra-articular fractures.^6^ The fractured patients may show clinical symptoms of ankle swelling, pain, subcutaneous ecchymosis, valgus deformity, movement disorder, etc., and there is a high risk of complicating with ankle traumatic arthritis, fracture malunion and other disorders.^7^ Previous research has documented that surgery is quite effective for periarticular ankle fractures, showing a prominently positive effect on accelerating the recovery of ankle function.^8^ While other scholars also reported that the peak of swelling occurred 1-3 days after the operation of periarticular ankle fracture, and the swelling of the affected part would produce negative impacts on the speed of wound healing and local blood circulation of the skin, reducing the effect of fracture healing, and lowing the quality of life of patients.^9^

Limb swelling and pain are common symptoms of patients after fracture surgery, which can be caused by multiple factors. It is mainly attributed to the exudation of soft tissues and blood vessels at the fracture site, which may obstruct blood supply and circulation at the affected area and aggravate the degree of swelling and pain.^7^,^10^ There are diverse types of fractures due to the relatively complex structure around the ankle joint. Meanwhile, there may be differences in the duration of recovery in patients with ankle fractures according to different diseases and different treatment methods. As evidenced by prior studies, periarticular ankle fracture has no obvious influence on the natural life span of patients.^11^,^12^ However, in the surgery, additional injuries may be induced by some operations, further leading to the swelling and pain of soft tissues at the fracture site. In this regard, postoperative supporting treatment is necessary to reduce postoperative pain and improve their quality of life.^13^

Clinically, Diclofenac Sodium Sustained-Release Capsules, Sodium Aescinate, mannitol and other drugs are generally applied for the treatment of patients with ankle fracture.^14^ In this study, mannitol was used in the postoperative treatment of patients with periarticular ankle fractures. In general, mannitol is a common choice for alleviating swelling in clinical treatment, which can improve the plasma osmotic pressure and effectively reduce the edema of patients. It has been revealed that rapid intravenous drip of 20% mannitol can effectively eliminate swelling and reduce the pain of affected limbs in the treated patients.^15^ Consequently, it can reduce swelling and prevent complications after fracture, which is conducive to early reduction and fixation of the fracture, lower hospitalization expenses and improve patient satisfaction.

Infrared ray, an invisible light, is an electromagnetic wave with a wavelength between microwave and visible light, which is called thermal ray by modern physics. The irradiated infrared can promote metabolism after being absorbed by the human body. Its mechanism is to resonate with molecules in the human body to promote microcirculation in vivo, so as to accelerate tissue regeneration of this part.^16^ Considering its advantage of strong penetration to produce a thermal effect after contact with skin, infrared ray is conducive to repairing the damaged blood circulation mechanism of patients. It has been recognized as one of the common clinical means to promote fracture healing. According to previous research, the thermal effect of infrared rays can not only strengthen cell viability but also quickly absorb metabolites and inflammatory components, which is effective to alleviate the swelling of the irradiated site and play an analgesic effect to some extent.^17^ Similarly, in this study, patients in the observation group received infrared irradiation, resulting in a significantly decreased degree of swelling and relieved pain of the patients.

Furthermore, Kinesiology tape, namely athletic patch, is mainly developed for joint treatment and pain relief. Currently, it is primarily used for treating joint diseases, relieving pain, improving circulation and reducing edema. It has the characteristics of elasticity and ultra-thin texture. It can be cut into different shapes according to the actual needs. In addition to fixation and protection roles, Kinesiology tape can also ensure the needs of human movement.^18^,^19^ Compared with traditional athletic patch, Kinesiology tape has the advantages of good elasticity, breathability and low sensitization.^20^ It has been demonstrated that postoperative application of Kinesiology tape after fracture surgery can promote deep lymphatic and blood circulation, and eliminate edema,^21^ which was also consistent with the results of this study.

### Limitations of this study:

It includes a small samplesize, with short follow-up time. Further intervention trials are needed to confirm these results.

## CONCLUSIONS

Infrared irradiation combined with mannitol and Kinesiology tape can effectively alleviate postoperative swelling and pain of patients with a periarticular ankle fracture. It has been recognized by patients and should be applied and promoted in clinical treatment.

### Authors’ Contributions:

**YL & TL:** Designed this study, prepared this manuscript, are responsible and accountable for the accuracy and integrity of the work.

**DG &** **XZ:** Collected and analyzed clinical data.

**BW &** **PL:** Data analysis**, s**ignificantly revised this manuscript.
